# Editorial: Artificial intelligence, machine learning, and data-mining techniques to increase cost-effectiveness in healthcare

**DOI:** 10.3389/fpubh.2024.1525628

**Published:** 2025-01-07

**Authors:** P. Wilner Jeanty, Marie-Rachelle Narcisse, Romain Crastes Dit Sourd

**Affiliations:** ^1^Data Science and Research Consulting, OhioHealth Corporation, Columbus, OH, United States; ^2^Department of Psychiatry and Human Behavior, The Warren Alpert Medical School, Brown University, Providence, RI, United States; ^3^Business School, University of Leeds, Leeds, United Kingdom

**Keywords:** artificial intelligence, machine and statistical learning, Markov models, quantile regression, patient care, hospital efficiency, healthcare, cost-effectiveness

Following the COVID-19 pandemic, healthcare systems all over the world have faced new barriers and challenges, such as increased patient volumes and staff shortages in delivering timely and high-quality care. Our Research Topic centers on exploring data science applications including artificial intelligence, machine learning, and data mining techniques to improve patient care while optimizing resource allocation. This Research Topic of Frontiers in Public Health includes six rigorously peer-reviewed articles offering actionable insights for healthcare providers and hospital administrators.

The first paper by Pei et al., entitled “*Cost-Effectiveness Analysis of Tumor Treating Fields for Lung Cancer Treatment in China*,” uses a Markov simulation model with a theoretical population of 276 patients metastatic non-small cell lung cancer (mNSCLC) over a 15-year time horizon to evaluate the cost-effectiveness of Tumor Treating Fields (TTFields) combined with standard-of-care for treating this cancer. The economic evaluation, including total costs, life-years, quality-adjusted life years (QALYs), and incremental cost-effectiveness ratio (ICER) values, reveals that the high costs of TTFields do not justify the modest survival benefits under current pricing models. This study aligns well with our Research Topic by applying a cost-effectiveness model providing insights into the economics of healthcare treatments. Future studies could combine simulation methods with AI techniques to optimize treatment decision models and dynamically update cost-effectiveness analyses as treatment parameters or patient demographics change. Additionally, research could use patient characteristics with mNSCLC to personalize these analyses using ML, AI, or DL analytics.

The next article by Liu et al. evaluates the cost-effectiveness of brigatinib and lorlatinib for treating ALK-positive non-small cell lung cancer (NSCLC), comparing the costs and outcomes of using brigatinib followed by lorlatinib before chemotherapy vs. after. The study uses past trial data to recompose individual patient-level data and estimate survival models, with cost figures derived from publicly available data and agent administration expenses. They authors employ Markov models to simulate disease progression, deriving transition probabilities between states, each with associated costs and quality-adjusted life years (QALYs). Their findings show that using brigatinib as a first-line treatment followed by lorlatinib is more cost-effective than using brigatinib as a second-line option. This approach improves QALYs and health outcomes while optimizing healthcare costs. The study supports a treatment strategy prioritizing first-line brigatinib to enhance the cost-effectiveness of NSCLC care.

In the third article titled ”*Demand prediction of medical services in home and community-based services for older adults in China using machine learning*“, Huang et al. use cross-sectional data from the 2018 Longitudinal Healthy Longevity Survey of 15,312 Chinese adults aged 65+ to predict demand for medical services in home and community-based services (HCBS) based on Anderson's behavioral model of health services utilization. Results from five predictive machine-learning methods (logistic regression, logistic regression with LASSO regularization, Support Vector Machine, Random Forest, and Extreme Gradient Boosting) unveiled that enabling factors, need factors, and behavioral factors were significant predictors of HCBS use. The authors argue that a robust conceptual model combined with rigorous machine learning methods could help better allocate limited medical resources to this segment of China's population with increasing demand for healthcare services. Future studies could employ machine learning or deep-learning methods based on longitudinal data to make causal inferences and integrate other dimensions of Andersen's Model of Health Services Use, such as social support, an enabling factor, to predict healthcare demand.

Entitled “*Hospital efficiency in the Eastern Mediterranean region: A systematic review and meta-analysis*”, the fourth article by Ravaghi et al. analyzes hospital efficiency in the Eastern Mediterranean, focusing on management's impact on healthcare quality and costs. Using a systematic review and meta-analysis of 37 studies, the authors evaluate factors like resource allocation, technology use, and hospital size through data envelopment analysis. They find significant heterogeneity across countries. Oman, for instance, has the highest mean technical efficiency among high-income nations and Bahrain the lowest. Among low- and middle-income nations, Iraq and Iran rank the highest, with Pakistan the lowest. Key factors affecting efficiency include internal structure, regional differences, and decision-maker participation in sustainability assessments. The authors recommend developing outpatient care, reducing supplier-induced demand, and strengthening hospital management and governance to improve efficiency and resilience against crises like COVID-19.

In the fifth article entitled “*The direct and indirect effects of length of hospital stay on the costs of inpatients with stroke in Ningxia, China*,” Su et al. analyze data from 129,444 ischemic stroke patients and 15,525 hemorrhagic stroke patients. Using quantile regression and structural equation models, they assess how length of hospital stay (LOHS) impacts hospitalization costs, considering indirect social factors. The study compares ischemic and hemorrhagic stroke patients, examining variables like age, hospital level, admission and discharge details, payment method, LOHS, and surgery. Findings show LOHS as the largest contributor to inpatient costs, with other factors such as the Charlson Comorbidity Index, payment type, surgery, hospital level, and discharge method also having significant direct and indirect effects.

Finally, the systematic review of 144 papers by Imani et al. identifies key indicators affecting hospital efficiency, categorized into input, process, and output indicators. Input indicators include hospital capacity, structure, and costs; process indicators cover quality-oriented processes and educational activities; and output indicators focus on activity-related and quality-related outcomes. The study emphasizes the importance of both quantitative and qualitative measures for a comprehensive analysis of hospital efficiency, providing a structured framework for healthcare providers and administrators to enhance patient care and optimize cost. Future research can build on this framework to develop predictive models for real-time decision-making and cost management, especially in response to dynamic hospital demands and potential pandemics.

The articles collectively highlight innovative applications of data science including machine and statistical learning techniques aiming at enhancing patient care and optimizing resource allocation in clinical and non-clinical settings. They provide actionable insights for healthcare providers and hospital administrators. They revolve around cost-effectiveness and efficiency and recommendations for improvements.

